# The Role of Sphingolipid Metabolism in Pregnancy-Associated Breast Cancer After Chemotherapy

**DOI:** 10.3390/biomedicines12122843

**Published:** 2024-12-13

**Authors:** Victor Blokhin, Tatiana Zavarykina, Vasily Kotsuba, Maria Kapralova, Uliana Gutner, Maria Shupik, Elena Kozyrko, Evgenia Luzina, Polina Lomskova, Darya Bajgazieva, Svetlana Khokhlova, Alice Alessenko

**Affiliations:** 1 Koltzov Institute of Developmental Biology of the Russian Academy of Sciences, Moscow 119334, Russia; victor.blokhin@hotmail.com; 2 Emanuel Institute of Biochemical Physics of Russian Academy of Sciences, Moscow 119334, Russia; tpalievskaya@yandex.ru (T.Z.); kapamariya97@gmail.com (M.K.); uliana.goutner@gmail.com (U.G.); mariashupik@gmail.com (M.S.); brenner123@mail.ru (P.L.); 3 B.I. Kulakov National Medical Research Center of Obstetrics, Gynecology and Perinatology of the Ministry of Health of the Russian Federation, Moscow 117997, Russia; lenochka525@gmail.com (E.K.); zhenyaluz@gmail.com (E.L.); ruzha88@mail.ru (D.B.); svkhokhlova@mail.ru (S.K.); 4 Federal Research Center “Fundamentals of Biotechnology” Russian Academy of Sciences, Moscow 119334, Russia; vasilyk@yandex.ru; 5 Department of Theoretical and Applied Chemistry, Federal State University of Education, Moscow 105005, Russia

**Keywords:** gene of sphingolipid metabolism, ceramides, sphingosine-1-phosphate, breast cancer, pregnancy, chemotherapy

## Abstract

Background: The aim of our study was to determine the role of sphingolipids, which control proliferation and apoptosis, in the placenta of pregnant women with pregnancy-associated breast cancer (PABC) after chemotherapy compared with healthy patients. Methods: We analyzed (by the PCR method) the gene expression of key sphingolipid metabolism enzymes (sphingomyelinases (SMPD1 and SMPD3), acid ceramidase (ASAH1), ceramide synthases (CERS 1–6), sphingosine kinase1 (SPHK1), sphingosine-1-phosphate lyase 1 (SGPL1), and sphingosine-1-phosphate receptors (S1PR1, S1PR2, and S1PR3)) and the content of subspecies of ceramides, sphingosine, and sphingosine-1-phosphate in seven patients with PABC after chemotherapy and eight healthy pregnant women as a control group. Results: We found a significant increase in the expression of genes of acid ceramidase (ASAH1), sphingosine-1-phosphate lyase 1 (SGPL1), sphingosine kinase (SPHK1), and ceramide synthases (CERS 1-3, 5, 6) in the samples of patients with PABC during their treatment with cytostatic chemotherapy. The increase in the expression of the enzymes’ genes was not accompanied by changes in the content of the studied sphingolipids. Such significant changes in the expression of genes controlling the level of CER, sphingosine, and S1P may indicate their ability to initiate the metabolism of pro-apoptotic and anti-apoptotic sphingolipids in the placenta of pregnant women with cancer undergoing chemotherapy in order to maintain levels typical of the placenta of healthy women. Conclusions: Our results may indicate the promising mechanism of placenta protection during chemotherapy for pregnant women with breast cancer and, consequently, of the newborn. This protective effect of the placenta and especially for the newborn has been discovered for the first time and requires more careful study.

## 1. Introduction

Sphingolipids are now recognized as multifaceted mediators in cancer biology and therapeutics [1,2,3,4,5,6,7,8,9]. Therefore, they may become novel targets for therapeutic applications.

Sphingolipids involved in the regulation of apoptosis and cell division, as well as enzymes and receptors that control their metabolism and functional activity are targets for anti-tumor therapy.

**Figure 1:**
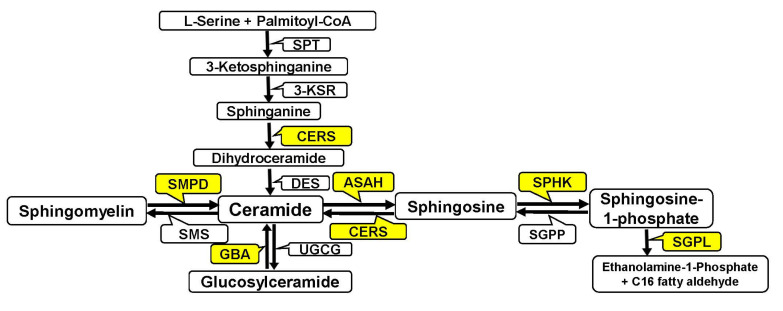
Abbreviated scheme of sphingolipid metabolism. SPT—serine palmitoyltransferase (the first step in the biosynthesis of sphingolipids; catalyzes the condensation of serine and palmitoyl-CoA); 3-KSR–3-ketosphinganine reductase (catalyzes the reduction of 3-ketodihydro sphinganine to sphinganine); CERS—(dihydro)ceramide synthase (key enzyme in the lipid metabolism of eukaryotic cells; there are six ceramide synthases (CERS1-6); each of which synthesizes ceramides de novo with distinct acyl chain lengths); DES—dihydroceramide desaturase (catalyzes the formation of double bond in C4–C5 positions in dihydroceramide); SMPD—sphingomyelinase (converts sphingomyelin to ceramide); SMS—sphingomyelin synthase (generates sphingomyelin and diacylglycerol from phosphatidylcholine and ceramide); ASAH—ceramidase (cleaves ceramides into sphingosine and fatty acids); GBA—glucosylceramidase (hydrolyzes glucosylceramide to form ceramide and glucose); UGCG—(catalyzes the transfer of glucose from UDP glucose to ceramide to produce glucosylceramide); SPHK—sphingosine kinase (converts sphingosine to sphingosine-1-phosphate); SGPP—sphingosine-1-phosphate phosphatase (dephosphorylates intracellular sphingosine-1-phosphate); and SGPL—sphingosine-1-phosphate lyase (the final step of S1P degradation; catalyzes the irreversible cleavage of sphingosine-1-phosphate to yield ethanolamine-1-phosphate and C16 fatty aldehyde).

Some enzymes capable of enhancing ceramide synthesis and cleaving S1P have been shown to promote cancer cell death. These enzymes include the following sphingomyelinases (SMPD): lysosomal acid SMPD (SMPD1), magnesium-dependent neutral SMPD (SMPD2); ceramide synthase (CERS); sphingosine-1-phosphat kinase (SPHK); and sphingosine-1-phosphate lyase (SGPL) (Figure 1) [10,11,12,13,14].

Many studies have shown that SPHK1 activation can induce cancer cell migration and that the SPHK1/S1P axis enhances the metastatic potential of cancer cells [15,16,17]. The role of sphingolipids in pregnancy is also under active investigation [18,19,20]. Sphingosine-1-phosphate (S1P) is a multifunctional lipid that regulates numerous physiologic processes. S1P normally mediates protective cellular processes, whereas ceramide (CER) induces destructive processes in the placenta [18,20]. In normal pregnancy, these processes occur within their normal physiological balance, thus preserving the structure and function of the various cells that make up the placenta. Thus, the S1P/CER rheostat determines the fate and function of cells in the placenta [18]. Signaling by the bioactive sphingolipid S1P and its precursors are emerging areas in pregnancy research [20]. According to data from Fakhr et al. [18] circulating CER levels increase towards end of gestation, suggesting a physiological role in parturition, despite the maintenance of circulating S1P concentrations. Several studies have shown no changes in the expression of genes of sphingolipid metabolism during normal placental development [21,22,23,24]. However, high levels of circulating S1P and ceramides are correlated with pregnancy disorders such as mellitus, preeclampsia, diabetes, and cancer. The expression of placental and decidual enzymes metabolizing S1P, as well as S1P receptors, is impaired in pregnancy complications [18].

Current data indicate the promising potential of S1P and related sphingolipids to be considered potential candidates for both diagnostics and therapeutics in cancer and pregnancy disorder studies.

We also believe that determining the gene expression levels of the enzymes involved in the synthesis of CERs, sphingosines, and S1P, which control apoptosis, may be a tool for diagnosing obstetric complications and the efficacy of cancer treatment in pregnant patients.

It has now been established that the efficacy of many drugs used for cancer chemotherapy exhibit their therapeutic effects through effects on sphingolipid metabolism, in particular through the activation of enzymes that generate the proapoptotic metabolites CER and sphingosine, such as sphingomyelinases [25] and ceramidase [1,4,25,26,27,28,29,30,31]. For example, platinum + taxane-based chemotherapy is associated with high response rates initially, but later these patients develop chemotherapy-resistant disease. All of these results suggest that CER can enhance the antitumor activity of docetaxel (DTX) in a synergistic manner, and the sphingolipid pathway involved in cancer development has also been found to contribute to chemoresistance and metastasis in a wide range of malignancies. In particular, the interaction between SPHK1 and S1PR1 has been shown to be altered during ovarian and breast cancer development in a variety of ways and therefore may be an attractive therapeutic target [8,32]. Taxane-induced peripheral neuropathy (TIPN) is known to be a side effect that limits the effective dose of the drug in breast cancer treatment. Ganglioside monosialic acid (GM1) is known to have neuroprotective properties. The use of GM1 in combination with taxanes was found to reduce the severity and incidence of TIPN after four cycles of taxane-containing chemotherapy in breast cancer patients [33]. Currently, CER-based combination therapy is being used as a new therapeutic strategy. The synergistic effect of CER in combination with DTX (CER + DTX) and the mechanism of this synergism have been studied. It was shown that simultaneous administration of CER and DTX in a molar ratio of 0.5:1 could induce an optimal synergistic effect on murine malignant melanoma cells (B16, CI = 0.31) and human breast carcinoma cells (MCF-7, CI = 0.48), suggesting that combined application prospects of CER + DTX treatment are promising [34].

CER glycosylation, through glucosylceramide synthase (GCS), allows for cellular escape from ceramide-induced programmed cell death. This glycosylation event confers cancer cell resistance to cytotoxic anticancer agents. It has been shown that multidrug resistance can be increased over baseline and then completely reversed in human breast cancer cells by GCS gene targeting [34,35]. The S1P/S1PR axis is considered a therapeutic target for cancer, particularly for breast cancer [34,35,36,37,38]. New drugs that directly affect sphingolipid metabolism are currently attracting attention. The best known is fingolimod (FTY720). This drug is currently used for the treatment of multiple sclerosis. As a sphingosine analog, FTY720 has been shown to affect S1P metabolism. Due to this property, it has a protective effect in many preclinical models of breast cancer [36,37,38]. Treatment with FTY720 has potentiated the anticancer effects of doxorubicin in different mouse models. Sarah Spiegel’s paper details the molecular mechanism of the antitumor action of FTY720 [39,40]. The protective effects of FTY720 are not limited to suppression of breast tumor development and progression only but are also seen in adjuvant therapy. In general, these studies show that FTY720 is a multifaceted drug that can work as an effective anticancer agent in its own right, as well as an adjuvant to hormonal therapy, conventional chemotherapy, and even radiation therapy for the treatment of not only estrogen-receptor-positive tumors but also more complex triple-negative breast cancer, as well as tumors that develop resistance to chemotherapeutic drugs [36,37,38,39,40,41].

## 2. Materials and Methods

### 2.1. Chemicals

All lipid analytical standards were obtained from Avanti (Avanti Polar Lipids, Birmingham, AL, USA). LCMS-grade acetonitrile and formic acid were from Fisher (Fisher Chemicals, Pittsburgh, PA, USA). LCMS-grade methanol was provided by EVA Science (EVA Science, Saint Petersburg, Russia). LCMS-grade isopropanol was procured from Panreac (Panreac Quimica, Barcelona, Spain). Ultrapure water was produced by a Millipore (Merck Millipore, Burlington, MA, USA), Direct-Q 3 system.

### 2.2. Study Population

This study was conducted in compliance with the principles of voluntariness and confidentiality in accordance with the Federal Law “On the Fundamentals of Protecting the Health of Citizens in the Russian Federation” and the 1964 Declaration of Helsinki and its subsequent amendments. This study was approved by the Biomedical Research Ethics Committee of Kulakov National Medical Research Center of Obstetrics, Gynecology and Perinatology of the Ministry of Health of Russia. Informed consent was obtained from each of the participants included in this study. The studied biological samples of patients with pregnancy-associated breast cancer (PABC) and healthy pregnant persons were obtained from Kulakov National Medical Research Center of Obstetrics, Gynecology and Perinatology of the Ministry of Health of Russia.

The criteria for the inclusion of patients in this study were a morphologically confirmed diagnosis of PABC; satisfactory general condition; normal function of hematopoiesis, kidneys, and liver; and the absence of metastasis. The diagnosis was established by histological examination.

The patient group included 7 patients with breast cancer. Seven patients with PABC received cytostatic chemotherapy during pregnancy (Table 1). All patients had live births delivered by spontaneous labor or cesarean section. Pre-term labor was observed in two patients. The median week of gestation at cancer diagnosis was 20 (min 4–max 26). On average, 3 (min, 1; max, 7) cycles of chemotherapy were applied. In this work, the samples of placenta from 7 women with PABC and 8 samples of placenta from healthy women were studied. The median age in the control group (healthy pregnant women) was 27.5 years old (min 23–max 39). In patients with PABC, the following obstetric parameters were analyzed: neonatal weight and height, fetal percentile (ultrasound data), placental weight, placental weight percentile (ultrasound data), and their relations with the expression of sphingolipid metabolism genes using logistic regression and Spearman’s rank correlation methods.

Samples of placenta were collected during labor in IntactRNA reagent (Evrogen, Moscow, Russia) for RNA protection. For lipid extraction, samples of placenta were collected in clear tubes. All samples were stored at −80 °C.

### 2.3. RNA Isolation, Reverse Transcription, and Real-Time PCR

RNA was isolated using Extract RNA reagent (Evrogen, Russia), and total RNA was subsequently cleaned by CleanRNA Standart Kit (Evrogen, Russia). Samples of RNA were stored with RNAse inhibitor RiboCare (Evrogen, Russia). For reverse transcription, an MMLV RT kit (Evrogen, Russia), random (dN)10 primer, and 1 µg of RNA were used. For the analysis of gene expression, real-time PCR was carried out using 5X qPCRmix-HS SYBR kit (Evrogen, Russia) on LightCycler96 (Roche, Vaud, Switzerland). Sequences of primers are presented in Table 2; primers were designed using Primer-BLAST. PCR conditions: denaturation phase—95.0 °C;—10 min; amplification phase—40 cycles; denaturation: 95.0 °C—10 s., amplification: Tann—annealing temperature—60.0 °C—60 s; melting curve: 95.0 °C—10 s., 65.0 °C—60 s., 97.0 °C—1 s. (single).

Calculation of relative expression was performed using the 2ΔΔCt method, normalized by housekeeping gene B2M.

### 2.4. Mass Spectrometry of Sphingolipids

Lipids were extracted from the placenta of healthy pregnant women and those with pregnancy-associated breast cancer after chemotherapy according to the Bligh and Dyer method [42]. Lipid analysis was performed by HPLC/MS on an Agilent 1290 Infinity system coupled to an Agilent 6460 triple quadrupole mass spectrometer (Agilent Technologies, Santa Clara, CA, USA).

A Phenomenex (Phenomenex Inc., Torrance, CA, USA) Luna Omega PS chromatographic column (50 × 2.1 mm, 1.6 µ) was used for the separation of lipids under the following conditions: 0.1% formic acid in water as mobile phase A; a mixture of methanol, acetonitrile, and isopropanol (4:1:1) containing 0.1% formic acid as mobile phase B; mobile phase flow–0.4 mL/min; and column temperature of 35 °C. The mobile phase gradient program was as follows: 60% B → 100% B in 8 min, hold at 100% B for 4 min, back to 60% B in 0.1 min, and re-equilibration for 2.9 min.

The electrospray ion source operated at a nebulizing gas pressure of 30 psi, whereas the drying gas flow and temperature were 10 L/min and 300 °C, respectively. The nebulizing gas, drying gas, and collision gas was nitrogen. Multiple reaction monitoring (MRM) mode was used for data acquisition. Positive ions only were detected. Ceramides exhibited two MRM transitions, resulting in a *m*/*z* 264.2 fragment, with the parent ions being [M + H]^+^ (used as quantifier) and [M − H_2_O + H]^+^ (used as qualifier). Sphingosine and sphingosine phosphate exhibited only single MRM transitions; sphingosine molecules, like the ceramides, produced the *m*/*z* 264.2 product ions, whereas sphingosine phosphate predominantly underwent dehydration. The corresponding values of optimized fragmentor voltage, collision energy, dwell time, and capillary voltage for each MRM transition are listed in Table 3. The cell accelerator voltage was set to 5 V for all transitions.

Each lipid was quantified using the external standard method. Calibration curves were obtained for the following concentration ranges: 5–10,000 ng/mL.

### 2.5. Statistical Analysis

Statistical analysis was carried out using Statistica 8.0.360.0 (StatSoft). Comparative analysis of gene expression and mass spectrometric analysis of sphingolipids was carried out in groups of patients with PABC and healthy women. The nonparametric Mann–Whitney test was used to compare the control and experimental groups. Differences were considered statistically significant at *p* < 0.05.

## 3. Results

### 3.1. Analysis of Gene Expression of Enzymes of Ceramide and Sphingoid Base Metabolism (Sphingosine and S1P) in Pregnancy-Associated Breast Cancer After Treatment

Breast cancer is the most common cancer in women, often leading to death. Because of this, intensive research is being conducted on causes, progression, and effective treatments for breast cancer. Sphingolipids have been found to be involved in many processes important in breast cancer, including growth, progression, transformation, and metastasis. PABC is the most common variant of cancer in pregnancy. In our study, we analyzed the gene expression of enzymes that are involved in the metabolism of ceramide and S1P, which largely determine apoptosis and cell division during breast cancer development.

We performed PCR analysis of the gene expression of enzymes involved in the generation of ceramides and S1P in placenta samples of patients with PABC after cytostatic chemotherapy with taxanes, chloroethylamines (cyclophosphamide), and anthracyclines in different combinations (Table 1). The results were compared to those of healthy pregnant patients.

#### 3.1.1. Analysis of Sphingomyelinase Gene Expression in Pregnancy-Associated Breast Cancer After Treatment

**Figure 2:**
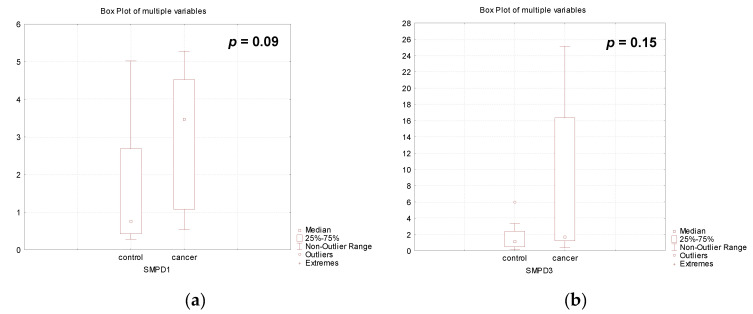
Gene expression of (**a**) acid (*SMPD1*) and (**b**) neutral (*SMPD3*) sphingomyelinases in the placenta of healthy pregnant women (control) and women with pregnancy-associated breast cancer after chemotherapy (cancer).

In our study, the tendency to undergo activation of the acidic sphingomyelinase gene was observed (4.5 times, *p* = 0.09, Figure 2b), whereas the neutral sphingomyelinase gene was slightly activated (1.5 times, *p* = 0.15, Figure 2a) during pregnancy in the course of breast cancer treatment (Figure 2).

#### 3.1.2. Analysis of Ceramide Synthase Gene Expression in Pregnancy-Associated Breast Cancer After Treatment

**Figure 3:**
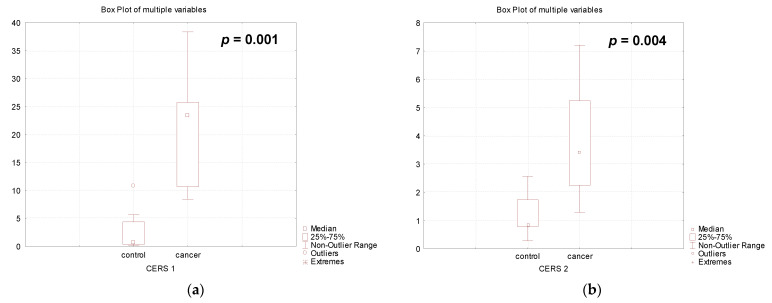

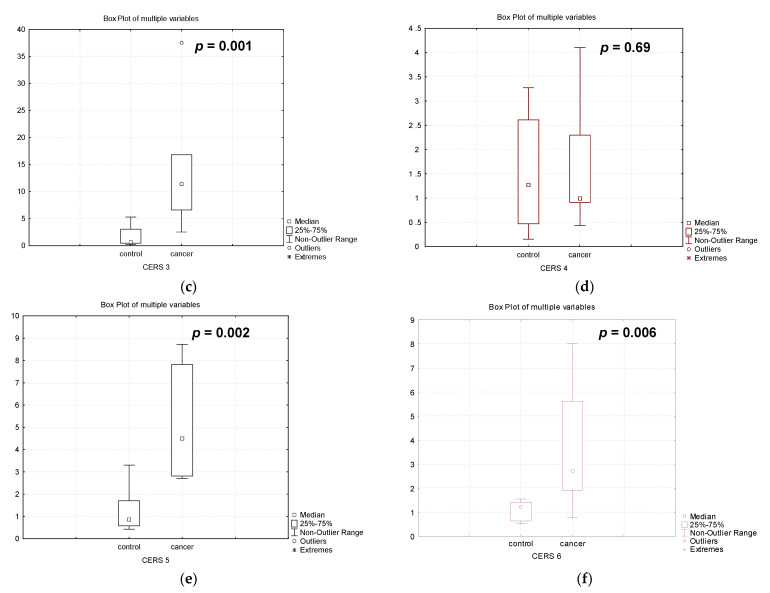
Gene expression of ceramide synthases in the placenta of healthy pregnant women (control) and women with pregnancy-associated breast cancer after chemotherapy: (**a**) *CERS1*, (**b**) *CERS2*, (**c**) *CERS3*, (**d**) *CERS4*, (**e**) *CERS5*, (**f**) *CERS6*.

When analyzing the expression of genes encoding different CERS isoforms, which are responsible for de novo synthesis of CER, we found a significant increase in the expression of the 0*CERS1*, *CERS2*, *CERS3*, and *CERS5* genes (*CERS1* (39.8-fold, *p* = 0.001), *CERS2* (3.9-fold, *p* = 0.004), *CERS3* (17.3-fold, *p* = 0.001), *CERS5* (5.1-fold, *p* = 0.002), *CERS6* (2.2-fold, *p* = 0.006)), except *CERS4* (no changes, *p* > 0.05), in the placenta of patients with PABC after their treatment compared to the results obtained for healthy pregnant women (Figure 3).

Sphingomyelinases and ceramide synthases act in opposite directions, and, depending on their activity, the content of total CER can both accumulate and decrease. In cases of balanced activity of these enzymes, its content can remain within the normal range. To determine the final effect of the gene expression, we studied the changes in the content of molecular types of CERs in the placenta of healthy women and patients after breast cancer therapy.

#### 3.1.3. Analysis of Acid Ceramidase (ASAH1) Gene Expression in Pregnancy-Associated Breast Cancer After Treatment

**Figure 4:**
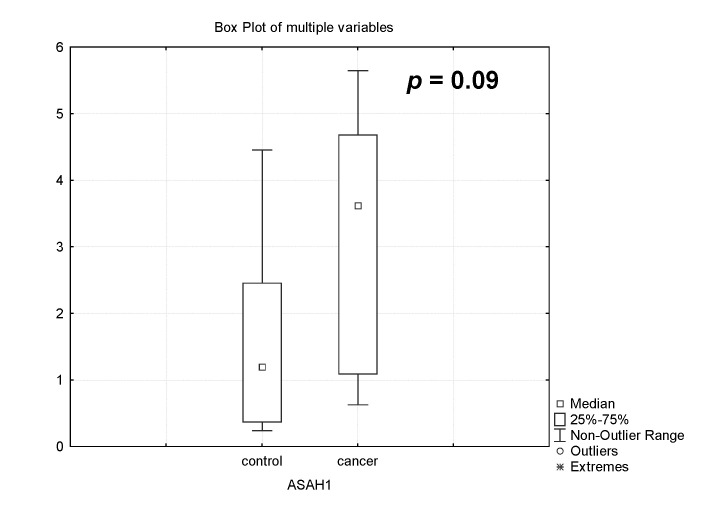
Gene expression of acid ceramidase (*ASAH1*) in the placenta of healthy pregnant women (control) and women with pregnancy-associated breast cancer after chemotherapy (cancer).

In our experiments, it was found that the expression of the acid ceramidase (*ASAH*) gene sharply increased during the chemotherapy of patients with PABC 3 times, *p* = 0.09 (Figure 4). This fact determined a possible decrease in the content of CER, which was formed by the degradation of sphingomyelin and its synthesis from sphingosine.

### 3.2. Analysis of Ceramide Species Level in the Placenta of Healthy Pregnant Women and Women with Pregnancy-Associated Breast Cancer After Chemotherapy

**Figure 5:**
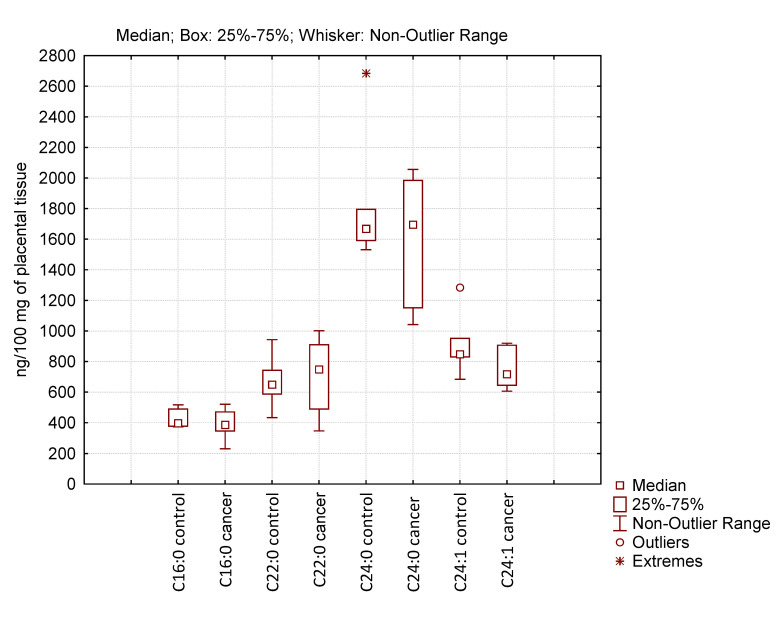
Level of ceramide species in the placenta of healthy pregnant women (control) and women with pregnancy-associated breast cancer after chemotherapy (cancer). Non-significant changes (*p* > 0.05) are not indicated.

We analyzed long-chain CER (C16:0, C22:0, C24:0, and C24:1), which are generally cytotoxic and pro-apoptotic, in the placenta of healthy pregnant women and women with PABC after chemotherapy (Figure 5).

Cytostatic chemotherapy drugs are able to induce the accumulation of CERs that lead to cancer cell death. Their effectiveness due to the increase in CER levels by increasing the expression of ceramide synthase genes results in the increased content of proapoptotic products of sphingolipid metabolism, CER. This effect of cytostatic chemotherapy drugs has been confirmed in other studies [5,43].

But when this proapoptotic agent is destroyed as a result of the more active action of ceramidase—in our case, acidic ceramidase—there can be either a decrease in its content or it can remain within the control values. When analyzing the content of the most significant molecular CER species in the placenta of healthy pregnant women and patients with PABC after chemotherapy by mass spectrometry, we found the same level for almost all CER species in these two groups of patients. This may imply the existence of a molecular mechanism to protect the placenta, and consequently the fetus, from the toxic effects of chemotherapy.

### 3.3. Changes in the Expression of Genes Controlling S1P Metabolism and Level of Sphingosine-1-Phosphate in the Placenta of Healthy Pregnant Women and Women with Pregnancy-Associated Breast Cancer After Chemotherapy

In our work, we investigated the expression of sphingosine kinase 1 (SPHK1) and sphingosine phosphate lyase 1 (SGPL1) genes that determine the metabolism of S1P, which is involved in pregnancy and malignancy and determines the efficacy of chemotherapy.

Sphingosine can be converted under the action of sphingosine phosphate kinase into anti-apoptotic S1P, which has anti-apoptotic properties, promotes cell proliferation, survival and migration, and participates in differentiation, neurogenesis, and angiogenesis [43,44]. Two main SPHK isoforms, types 1 and 2, have been identified and described; these isoforms have a similar polypeptide structure but differ in cellular localization and physiological functions. SPHK1 is the most studied enzyme of S1P metabolism. The basal level of SPHK1 activity maintains the cellular balance of sphingosine and S1P under the action of several agonists on the cell, including proinflammatory cytokines, various growth factors, etc. By contrast, sphingosine phosphate lyase 1 (*SGPL1*) degrades S1P.

**Figure 6:**
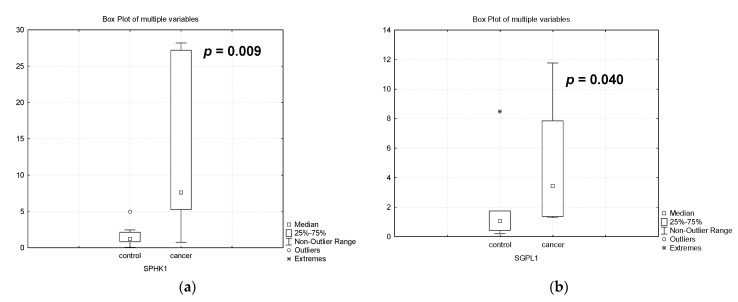
Gene expression of sphingosine kinase 1 (*SPHK1*) (**a**) and sphingosine phosphate lyase 1 (*SGPL1*) (**b**) in the placenta of healthy pregnant women (control) and women with pregnancy-associated breast cancer after chemotherapy (cancer).

In our experiments, we found a dramatic increase in *SPHK1* gene expression in patients with PABC undergoing treatment compared to healthy patients (by 6.3 times, *p* = 0.009, Figure 5). Despite the fact that S1P generated by SPHK1 has anti-apoptotic properties, it can influence survival through its effects on drug resistance [12]. Simultaneously, there is a sharp activation of the expression of the sphingosine phosphate lyase gene (4 times, *p* = 0.040, Figure 6). This fact determines the possible degradation of S1P. Consequently, two opposing processes occur simultaneously that may maintain a stable level of S1P in the placenta. This fact determines the possible degradation of S1P.

#### 3.3.1. Analysis of Sphingosine and Sphingosine-1-Phosphate Level in the Placenta of Healthy Pregnant Women and Women with Pregnancy-Associated Breast Cancer After Chemotherapy

**Figure 7:**
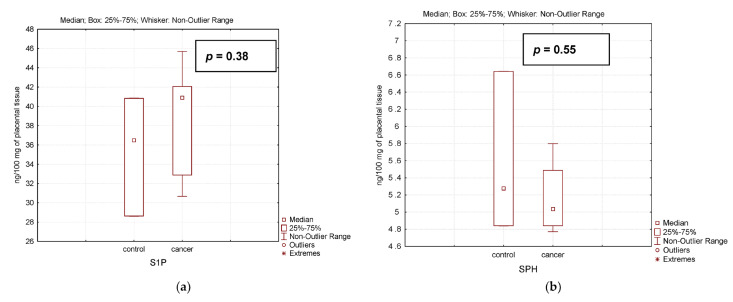
Level of sphingosine-1-phosphate (S1P) (**a**) and sphingosine (SPH) (**b**) in the placenta of healthy pregnant women (control) and women with pregnancy-associated breast cancer after chemotherapy (cancer).

The analysis of sphingosine and sphingosine-1-phosphate (Figure 7) content in the placenta of healthy pregnant women and women with PABC after chemotherapy has shown that these sphingolipids, which have opposite properties in cell life, also remain stable after chemotherapy. Simultaneous activation of the gene expression of genes with opposite properties has a stabilizing effect on the content of these sphingolipids. These facts additionally confirm the possible existence of a very reliable mechanism of placental protection against the toxic effect of chemotherapy.

#### 3.3.2. Analysis of Sphingosine-1-Phosphate Receptors (S1PR1, S1PR2 and S1PR3) Gene Expression in Pregnancy-Associated Breast Cancer After Treatment

S1P can bind to one of five specific G protein-coupled cell surface S1P receptors (S1PR1-5). S1PR1 and S1PR3 have been linked to breast cancer progression. The SPHK1-S1PR1 axis has been shown to be altered in different localizations of cancer in multiple ways and therefore represents an attractive therapeutic target [45,46,47,48].

**Figure 8:**
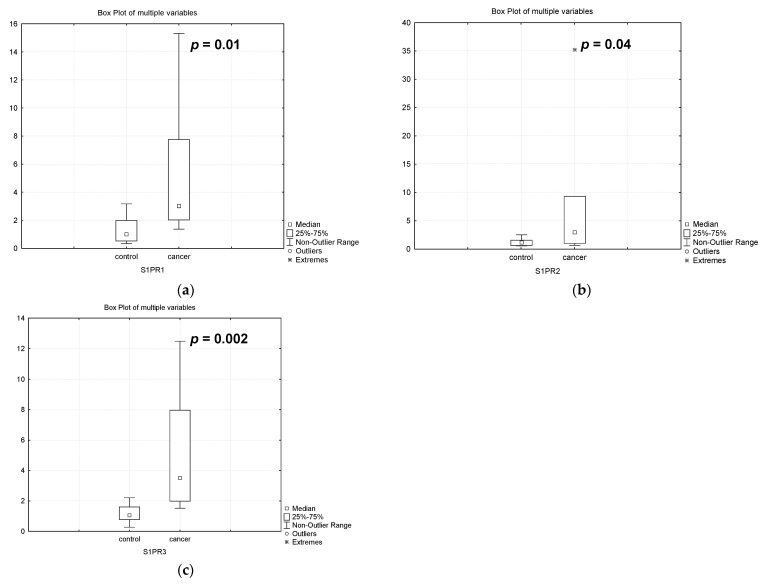
Gene expression of sphingosine-1-phosphate receptors in the placenta of healthy pregnant women (control) and women with pregnancy-associated breast cancer after chemotherapy (cancer): (**a**) *S1PR1*, (**b**) *S1PR2*, (**c**) *S1PR3*.

Our experiments show that the gene expression of these two receptors is significantly higher in patients with PABC undergoing treatment than in controls: *S1PR1* 3 times, *p* = 0.01; *S1PR2* 2.6 times, *p* = 0.04; and *S1PR3* 3.3 times, *p* = 0.002 (Figure 8).

### 3.4. The Effect of PABC and Therapy on the Newborn and Mother

No differences were found between the healthy pregnant women and patients with PABC in the following obstetric parameters studied: newborn weight (*p* = 0.23), newborn height (*p* = 0.28), fetal percentile (*p* = 0.54), and placental weight (*p* = 0.69). Among the newborns in the group of patients with PABC, there was a higher frequency of pathological conditions in the early neonatal period; most newborns had a combination of several such conditions (5/7, 71.4%). However, no severe malformations were identified. Physiological birth tumor was observed in 3/7 (42.9%) newborns; jaundice in 2/7 (28.6%); anemia in 2/7 (28.6%); interatrial communication in 2/7 (28.6%) newborns; and thermoregulation disorder in 2/7 (28.6%). One of newborns in this group (1/7, 14.3%) was in a severe condition, accompanied by depression of the central nervous system and respiratory distress syndrome, anemia, the presence of a patent ductus arteriosus, and interatrial communication. This condition might have been due to premature labor at 36 weeks and 3 days. In children from women with PABC after chemotherapy in 1/7 (14.3%) cases, a “felt hat” type of ossification of the skull bones was recorded. In 1/7 (14.3%) cases, the newborn had rhinitis, otitis, and toxic erythema.

In children from the group of healthy pregnant women, only a birth tumor was observed in 2/7 (28.6%) newborns (Table 4). No differences were found in this parameter between the groups of newborns from healthy pregnant women and women with PABC (*p* = 0.46).

Thus, the frequency of pathological conditions differed in newborns of the PABC group (median M = three pathologies in the newborn) from newborns in the group of healthy pregnant women (median M = zero pathologies in the newborn, *p* = 0.009).

We excluded from the analysis physiological birth tumors as an insignificant condition, often considered a normal variant, and jaundice, which is characteristic of the physiological process of a child’s adaptation to a new environment and is often observed in the neonatal period as a norm. Differences between groups in the frequency of pathological conditions in newborns remained, median M = 1 in women with PABC and M = 0 in healthy pregnant women, *p* = 0.0001.

It should be taken into account that healthy patients without any serious acute or chronic diseases before and during pregnancy were selected as the control group. In the general population of Russia, the incidence of neonatal pathological conditions is observed in 43.1% of children [49]. In our work, they were detected in 6/7 (86%) newborns in the PABC group. However, the spectrum of pathologies detected did not allow for their classification as severe conditions, with the exception of the child of patient BC5. According to the literature, children of patients with PABC are characterized by fetal growth restriction, premature labor, low birth weight, stillbirth, and increased neonatal mortality [50]. In our work, from the listed conditions above, only premature labor was observed in 3/7 patients with PABC (42.9%), which was not revealed in the group of healthy pregnant women, *p* = 0.020.

Thus, in the PABC group, no increase in the incidence of severe neonatal pathologies was detected, but an increase in the incidence of premature labor was observed. However, at this stage, it is impossible to assert if this was an effect of chemotherapy. More extensive studies are needed due to the fact that premature labor is a common obstetric complication of pregnancy [51,52].

During further follow-up over 12 months, the subsequent growth and development of children born from mothers with PABC proceeded without any special features. All of the children had no health problems at the time of the cut-off. Among the mothers in the PABC group, two of them (BC4, BC5) showed progression of the disease, whereas the rest were in remission after one year of follow-up. Patient BC4 had metastases in the brain. Patient BC5’s had rapidly progressing, multiple distant metastases in the liver, lungs, and pleura, in addition to greater omentum.

The data available at this stage are insufficient to assess the association between the expression of sphingomyelin metabolism genes in the placenta and the effectiveness of the treatment of PABC. This requires clarifying the commonality of sphingolipid metabolism in the body of pregnant women and the admissibility of extrapolating expression data in the placenta to the body level.

In the PABC group, cytotoxic chemotherapy was used, with 5/7 (71.4%) patients receiving doxorubicin in the chemotherapy regimen. No association was found between the presence of doxorubicin in the chemotherapy regimen and the following obstetric parameters: newborn weight (*p* = 0.28), newborn height (*p* = 0.18), fetal percentile (*p* = 0.74), placental weight (*p* = 0.73), and placental percentile (*p* = 0.20). Patients who did not receive doxorubicin had lower levels of CERS4 and SMPD3 gene expression in the placenta. For the CERS4 gene, the median expression in the group receiving doxorubicin was two-fold higher compared to patients who did not receive this drug, *p* = 0.021. For the SMPD3 gene, expression in the doxorubicin group increased by 12.7 times; however, differences were observed at the trend level, *p* = 0.071.

## 4. Discussion

Since the aim of our study was to determine the role of sphingolipid metabolism genes and the products that control proliferation, apoptosis, and the efficacy of cancer chemotherapy by changing their expression level in the placenta of pregnant women against the background of malignant neoplasm development, we examined changes in the expression of key genes of CER and S1P metabolism and its level, which may play an important role both in the development of oncology and pregnancy. In addition, they reflect the efficacy of chemotherapy and signal the emergence of resistance to many drugs used to treat cancers of various localizations. The S1P/CER ratio determines cell fate because S1P is protective, as opposed to long-chain CERs (C16:0-C24:0) that are generally cytotoxic and pro-apoptotic [46].

The expression of acidic and neutral sphingomyelinase genes produces CERs from sphingomyelins. CERs are also produced through de novo synthesis in the endoplasmic reticulum. CERs can also be metabolized by acidic, alkaline, and neutral ceramidases to yield sphingosine [47], which is then phosphorylated by SPHK to produce S1P.

Since S1P is formed as a product of CER metabolism, the S1P/CER ratio is highly dependent on the level and activity of enzymes regulating S1P and CER turnover. The expression of enzymes synthesizing and degrading S1P controls its levels in the placenta, and decidua in normal pregnancy is positively correlated with increasing gestational age [18,20]. This suggests an increase in S1P turnover with the course of pregnancy, but whether S1P levels in the placenta change with increasing gestational age remains unknown. In complicated pregnancy, the expression of placental S1P-producing enzymes is decreased, and S1P-degrading enzymes is increased.

Because S1P acts in balance with CERs, their ratio determines cell fate and function in the placenta [18]. CERs induce apoptosis, and high levels of circulating CERs are often associated with pregnancy pathologies [18]. Current evidence suggests that S1P and CERs are promising for being considered strong candidates for both diagnosis and therapy in future studies. Sphingolipids also reflect the development of malignant neoplasms [4,6,7,26,36,45,48].

In our case, we paid special attention to patients with PABC, which is the most frequent of all pregnancy-associated cancers. Moreover, breast cancer is the first cause of cancer mortality and incidence among women. We found significant changes in the expression levels of genes controlling CER and S1P synthesis in pregnant patients treated with chemotherapy for breast cancer. The expression levels of sphingomyelinases and ceramide synthases, responsible for the level of CER content in the placenta, dramatically increased.

Mass-spectrometric analysis of CERs showed unchanged levels in groups of healthy pregnant women and patients with PABC. Activation of sphingomyelinase, sphingomyelin synthase, and ceramidase gene expression can lead to the synthesis of corresponding enzymes with opposite properties that can be activated. It is this process that may keep CER levels in patients with PABC at control levels. It is possible that through this mechanism the placenta protects the fetus from the toxic effects of chemotherapeutic agents. We plan to investigate this question in the future.

The expression of the *SGPL* gene is dramatically increased during chemotherapy of patients with PABC. However, we also found a dramatic increase in *SPHK1* gene expression in patients with PABC undergoing treatment compared to healthy patients. Thus, we showed that the genes of two enzymes with opposite properties in the process of S1P metabolism are activated in the placenta of pregnant women with breast cancer after chemotherapy compared to healthy pregnant women. It is these processes that can explain the maintenance of S1P content in these patients at the same level as in healthy women. In other words, our experiments show that in the placenta of pregnant women with breast cancer after chemotherapy, the balance of CERs and S1P, characteristic of healthy pregnant women, is preserved.

The special role of S1P is determined by the fact that it can bind to one of five specific S1P receptors on the cell surface. Two receptors, S1PR1 and S1PR3, are associated with breast cancer progression. The SphK1/S1P/S1PR axis in breast cancer cells promotes their growth, survival, dissemination and metastasis. It is also usually accompanied by increased chemotherapeutic resistance [31]. In our experiments, we found increased expression of *S1PR1* and *S1PR3* in the placenta of women with PABC after chemotherapy. These results may indicate the development of resistance to the drugs used to treat breast cancer. However, at this stage of our studies, which are pilot studies, we cannot answer this question. Nevertheless, such significant changes in the expression of genes controlling the level of CERs, sphingosine, and S1P may indicate their ability to initiate the metabolism of pro-apoptotic and anti-apoptotic sphingolipids in the placenta of pregnant women with cancer undergoing chemotherapy in order to maintain levels typical of the placenta of healthy women. This hypothesis is supported by the fact that in the PABC patient group, no increase in the incidence of severe neonatal pathologies was detected. During monitoring over 12 months, the subsequent growth and development of children born from mothers with PABC proceeded without any special features, and all children had no health problems.

In conclusion, we may note that our results may indicate the promising mechanism of protection by placenta during the chemotherapy of pregnant women with breast cancer and, consequently, of newborns. This effect of protection by the placenta, especially, for the newborn has been discovered for the first time and requires more careful study.

## Figures and Tables

**Table 1:** Clinical characteristics of patients with pregnancy-associated breast cancer (PABC).

Parameter	Data
Age, median -Minimum–maximum	37 years old 31–43 years old
Stage of the disease: -II -III	-5 out of 7 (71.4%) -2 out of 7 (28.6%)
Luminal A Luminal B HER2-negative Luminal B HER2-positive Triple negative	-1 out of 7 (14%) -3 out of 7 (43%) -1 out of 7 (14%) -2 out of 7 (29%)
Chemotherapy: -Taxanes -Chloroethylamines, anthracyclines -Taxanes, chloroethylamines, anthracyclines -Taxanes, chloroethylamines	-1 out of 7 (14.3%) -4 out of 7 (57.1%) -1 out of 7 (14.3%) -1 out of 7 (14.3%)

**Table 2:** Primers for analysis of gene expression.

Gene, Number in NCBI	Sequence	Length, b.p.
*SPHK1* NM_001142602.2	F: GAGCAGGTCACCAATGAAG R: ATCAGCAATGAAGCCCCAG	150
*SGPL1* NM_003901.4	F: TTCCATTCCCCATCTCAGG R: CACACACACACACACACAC	240
*ASAH1* NM_001363743.2	F: AGTCAATAGCTTGTCTTCGTC R: GTGTTTACTGTCCCGTTACTC	265
*SMPD1* NM_001365135.2	F: AGTCAATAGCTTGTCTTCGTC R: GTGTTTACTGTCCCGTTACTC	265
*GBA1* NM_001171812.2	F: GCCACAGCATCATCACGAAC R: TAGCACGACCACAACAGCAG	293
*SMPD3* NM_018667.4	F: CCTTCATACCCACCACCTAC R: CAGAAGAGAAAGCCGAGAAAC	145
*CERS2* NM_022075.5	F: CACCCCATCCTCAATAACAAC R: CCTCTCACTTTCTCCTTTTTCC	148
*CERS1* NM_001387444.1	F: CCCCAAGCCTACTCCAAAAC R: AACTACTCCTCACCACCCAC	216
*CERS4* NM_024552.3	F: AGACCAGGAGGCAAGTGAAG R: CGAAGGAGGACAGGTAGAAGAG	225
*CERS6* NM_203463.3	F: AGGACAGGAGTGGACAAAG R: AGGGGAAAAGCGAGATAGAG	154
*CERS3* NM_001378789.1	F: GAAGAGGAAGAGGAAGAGGAAG R: TGGTGAGAAAGAGGGAAGGG	226
*CERS5* NM_147190.5	F: GCCCTTCCCATATCTACTCTTC R: GCACAAACGCACATCAAC	179
*S1PR1* NM_001400.5	F: AATTCAGCCGCAGCAAATC R: AACTCTACCCACCAACACCC	279
*S1PR2* NM_004230.4	F: TGTATGGCAGCGACAAGAG R: ACAGGATGATGGAGAAGATGG	192
*S1PR3* NM_005226.4	F: CCCACTCTTCATCCTCTTCC R: GCTGCTATTGTTGCTGCTG	268

Note: F—forward primer; R—reverse primer.

**Table 3:** Optimized parameters for the MRM transitions of the studied lipids.

Sphingolipid	MRM Transition	Fragmentor Voltage, V	Collision Energy, V	Dwell Time, ms	Capillary Voltage, kV
C16 Cer (d18:1/16:0) C22 Cer (d18:1/22:0) C24 Cer (d18:1/24:0) C24:1 Cer (d18:1/24:1(15Z)) Sph S1P	538.0→264.2 520.0→264.2 622.5→264.2 604.5→264.2 650.5→264.2 632.5→264.2 649.0→264.2 631.0→264.2 305.5→264.2 380.5→362.5	100 150 100 150 100 100 100 100 150 100	35 20 35 35 35 35 35 35 5 15	45 45 45 45 55 55	4000 4000 4000 4000 3000 3000

**Table 4:** Pathological conditions in newborns in groups of healthy pregnant women and patients with PABC (pregnancy-associated breast cancer).

Number of Patient	Pathological Condition	Number of Pathological Conditions in the Newborn
BC1 BC2 BC3 BC4 BC5 BC6 BC7 C1 C2 C3 C4 C5 C6 C7 C8	Physiological birth tumor, Rash on face Toxic erythema, rhinitis, otitis, jaundice Interatrial communication, thermoregulation disorder, asymmetry of the sizes of the lateral ventricles of the brain, birth tumor Ossification disorder of the skull bones Serious condition, respiratory distress syndrome, central nervous system depression, patent ductus arteriosus, interatrial communication, thermoregulation disorder, anemia No Jaundice, anemia, physiological birth tumor No No No Physiological birth tumor No Physiological birth tumor No No	2 4 4 1 7 0 3 0 0 0 1 0 1 0 0

Note: BC (breast cancer)—pregnancy-associated breast cancer (PABC) patients; C (control)—healthy pregnant women.

## Data Availability

The data presented in this study are available on reasonable request from the corresponding author.

## Abbreviations

ASAH1	acid ceramidase
GBA	glucosylceramidase
CDase	ceramidase
CER	ceramide
CER + DTX	CER in combination with docetaxel
C1P	ceramide-1-phosphate
CERS 1–6	ceramide synthases
DTX	docetaxel
FTY720	fingolimod
GCS	glucosylceramide synthase
MCF-7	human breast carcinoma cell
MRM	multiple reaction monitoring
PABC	pregnancy-associated breast cancer
S1P	sphingosine-1-phosphate
SPH	sphingosine
SGPL1	sphingosine-1-phosphate lyase 1
SMPD1	acidic sphingomyelinase
SMPD3	neutral sphingomyelinase
SMS	sphingomyelin synthase
SPHK1	sphingosine kinase1
